# Commentary: Why we should report results from clinical trial pilot studies

**DOI:** 10.1186/1745-6215-14-14

**Published:** 2013-01-11

**Authors:** Lawrence Friedman

**Affiliations:** 1 Rockville, MD, USA, MD, 0028, USA

## 

Should clinical trial pilot studies be reported? Thebane, Ma, Chu, et al. argue in their discussion of the conduct of pilot studies, that they should be published [1]. Others, such as the editorialist for *The Diabetes Educator*, take a more cautious approach to publishing pilot studies [2]. I agree with those who advocate publication. I might also note that a September 27, 2012 search of “randomized clinical trial pilot studies” in PubMed contained over 10,400 citations. Although some of these may not reflect individual pilot studies, there may, though, be many more (without “randomized” in the search term, the number is over 22,000).

First, I will define my terms. By a pilot study, I mean one that is conducted preliminary to a full-scale late phase (phase 3 or 4, in common terminology) trial. The pilot study is conducted in order to address questions important for the design and conduct of the full-scale trial, and is sometimes called a feasibility or vanguard study. Obviously, the usual early phase (phase 1 and 2) studies could serve those purposes. (The results of these studies are often not reported, and whether they should be is another issue.) But, often, even after the early phase studies are done, we still have questions about participant screening and enrollment, adherence over some period of time, administration of the intervention in community settings, outcome assessment, and the like. Pilot studies are designed to answer those sorts of questions that are essential in the development of the full-scale trial.

Pilot studies can also be of two sorts. One is an “internal pilot,” where, if it is successful and meaningful protocol changes (a phrase that is subject to judgment) are not made in the transition to a full-scale trial, it more or less seamlessly merges into the larger, definitive clinical trial. In that situation, the full-scale trial rules about reporting interim data would apply. An example is the pilot study that was done prior to the full-scale Antiarrhythmics Versus Implantable Defibrillators (AVID) trial in people who had been resuscitated from ventricular fibrillation or had had sustained ventricular tachycardia [3]. The pilot study of 200 participants showed that recruitment could be accomplished and that crossover would be tolerably low. As a result, the original 200 participants were included in the full-scale trial [4]. The second, and probably more common kind of pilot study, is one that is done separately from the full-scale trial. After the completion of the pilot study, the results are examined, and depending on those results, a decision is made whether and how to conduct the full-scale trial. The investigators need to decide, and need to persuade the prospective funder of the full-scale trial, that the pilot study is sufficiently encouraging that a full-scale trial should be conducted. The data from this kind of pilot study are typically not incorporated into the full-scale trial. One reason is that there may be sufficiently large differences in the protocols of the two trials. In addition, questions might be raised about the appropriateness of deciding whether or not to include data after a trend has or has not been observed. An example is the pilot study that preceded the full-scale Cardiac Arrhythmia Suppression Trial (CAST) [5]. This first study, the Cardiac Arrhythmia Pilot Study (CAPS) demonstrated that three of four anti-arrhythmic agents could successfully reduce ventricular arrhythmias with tolerable adverse effects over a one-year period [6]. Following this pilot study, the CAST was successfully implemented as an entirely separate trial.

What are the arguments for not reporting the results of such independent pilot studies? They are small and usually of shorter duration than the full-scale trial. The intervention, dose, method of administration of the intervention, and population studied may be somewhat different. How the outcomes are measured may be different. In other words, the results from the pilot study may not reflect the “true” effect of the intervention and could mislead readers.

Why would we want to report the results of pilot studies? First, even though the investigators who conduct pilot studies will know the results, and can use them in the design of their full-scale trial, the data might also be useful to others. It is the rare clinical trial that is researching a condition or type of intervention so special that such information would not be of benefit to others. Second, meta-analyses incorporate not just the data from the large, adequately powered trials, but from smaller trials, including pilot studies (as long as they were randomized). Third, almost all (maybe all) clinical research carries some risk to participants. If a study (even a pilot study) is important enough to put participants at some risk, the results should be as broadly distributed as possible. Finally, if the pilot study does not lead to a full-scale trial, it may be particularly important for other researchers to understand why it did not. What did not work, why didn’t it work, and is there a way it can be improved or corrected?

We need to keep in mind that most clinical trials are sponsored by industry. Despite the scientific and academic imperative to publish research findings, drug companies are rarely interested in providing information of use to their competitors. Therefore, they may be reluctant to publish the results of pilot trials. But, if these pilot trials were done by academically-based researchers, there could be a conflict between the needs of the sponsoring company and academic freedom and obligations. Regardless of the desire by industry to keep pilot study data confidential, at most there should be a delay in publication, not a failure of publication. Once the full-scale trial has been completed, or is sufficiently underway so that the sponsoring company has a lead over its competition, there is no longer a compelling commercial need to prevent publication of the pilot trial results.

I think that the arguments in favor of reporting outweigh those against doing so. Science is (or should be) open. Science and medicine advance best when there is easy and rapid sharing of findings. To be sure, all reports need to be accompanied by appropriate caveats, so that readers are not misled by results that are at best tentative and likely to change. But we need to have confidence that those who read, interpret, and use the reports are smart enough to understand the limitations. Journals such as this one can provide a great service by publishing the results of randomized pilot studies.
